# Meteorological Report for January, 1880

**Published:** 1880-02

**Authors:** 


### METEOROLOGICAL REPORT FOR JANUARY, 1880.

| RAIN. DATE | IN. | Mean daily Barometer. | Mean daily Thermometer | Mean daily Humidity. | Maximum Thermometer | Minimum Thermometer | Prevailing direction of Wind. | Maximum hourly velocity of Wind, (Daily.) |
|---|---|---|---|---|---|---|---|---|
| 1 | 0 | 30.321 | 62.2 | 71.3 | 69 | 54 | N. W. | ......... |
| 2 | 0 | 30 324 | 62 7 | 65.3 | 70 | 55 | N. W. | ......... |
| 3 | 0 | 30.361 | 62 0 | 66.0 | 69 | 55 | S. E. | ......... |
| 4 | 0 | 30 352 | 62 0 | 69.7 | 70 | 55 | S. E. | ......... |
| 5 | 0 | 30.274 | 63.0 | 66.7 | 71 | 56 | S. E. | ......... |
| 6 | 22 | 30.247 | 61 2 | 75.3 | 70 | 58 | S. E. | ......... |
| 7 | 10 | 30.206 | 63 0 | 85.0 | 68 | 58 | S. W. | ......... |
| 8 | 1.10 | 30.062 | 59.0 | 91.7 | 65 | 55 | E. | ......... |
| 9 | 01 | 30 082 | 51 2 | 90.7 | 55 | 50 | E. | ......... |
| 10 | 02 | 30 149 | 52 2 | 93 3 | 60 | 49 | S. E. | ......... |
| 11 | 02 | 30.195 | 62 7 | 86.7 | 67 | 57 | S. E | ......... |
| 12 | 20 | 30.154 | 62 2 | 87.3 | 70 | 59 | S W. | ......... |
| 13 | 06 | 30.319 | 43.5 | 50.7 | 62 | 38 | N. W. | ......... |
| 14 | 0 | 30.326 | 39 5 | 47.7 | 46 | 30 | E. | ......... |
| 15 | 0 | 30.245 | 47.0 | 60 3 | 56 | 34 | N. E. | ......... |
| 16 | 0 | 30 197 | 50.5 | 65.3 | 57 | 37 | E. | ......... |
| 17 | 03 | 30 038 | 54.7 | 73 0 | 63 | 41 | S. W. | ......... |
| 18 | 0 | 30 087 | 54.5 | 51 3 | 59 | 47 | N. W. | ......... |
| 19 | 0 | 30.073 | 60.0 | 43.0 | 68 | 48 | S. W. | ......... |
| 20 | 0 | 29 893 | 61.2 | 64.3 | 67 | 52 | S. W. | ......... |
| 21 | 0 | 29 794 | 59 2 | 80.3 | 64 | 53 | S. | ......... |
| 22 | 08 | 29.688 | 52.7 | 69.0 | 62 | 46 | S W. | ......... |
| 23 | 0 | 29 959 | 42.2 | 54 0 | 48 | 36 | N. W. | ......... |
| 24 | 0 | 30 120 | 40.7 | 54.0 | 50 | 32 | N E. | ......... |
| 25 | 0 | 30 041 | 39 5 | 84 3 | 45 | 32 | N E. | ......... |
| 26 | 83 | 29 854 | 40.0 | 76.3 | 43 | 36 | N. E | ......... |
| 27 | 09 | 29 845 | 54.5 | 81.3 | 62 | 40 | N. W. | ......... |
| 28 | 0 | 30 061 | 60.0 | 59 7 | 69 | 46 | N. W. | ......... |
| 29 | 0 | 30.232 | 59 7 | 57.0 | 67 | 51 | E. | ......... |
| 30 | 08 | 30.289 | 49 0 | 85.3 | 59 | 48 | E. | ......... |
| 31 | 02 | 30.115 | 54.2 | 84.7 | 62 | 48 | W | ......... |
| Sums | 2.86 | 933 903 | 1786.0 | 2199.5 | 19.13 | 15.56 | ......... | ......... |

Highest Barometer, 30.442, on the 14th.
Lowest Barometer, 29.575, on the 22nd.
Monthly range of Barometer, 0.867.
Highest Temp. 71°, on 5th. Lowest Temp. 30°, on 14th.
Monthly range of Temperature, 41°.
Greatest daily range of Temperature, 24°, on the 13th.
Lowest daily range of Temperature, 5°, on the 9th.
Mean of Maximum Temperatures, 61°7.
Mean of Minimum Temperatures, 51°9.
Mean daily range of Temperature, 9°8. Total rainfall, 2.86 inches.
Depth of unmelted snow lying on ground at end of month, 0.
Prevailing wind, N. W. Total movement of wind, 72.00 miles.
Maximum velocity of wind and direction, 29 miles per hour, N. E., 26th
Number of cloudy days on which no rain fell, 4.
Total number of days on which rain fell, 13.

H. HALL, Corp'l. Signal Service U.S. A.

Atlanta, Ga., Jan. 1, 1880.

